# Exact Inference for the Dispersion Matrix

**DOI:** 10.1155/2014/432805

**Published:** 2014-09-14

**Authors:** Alan D. Hutson, Gregory E. Wilding, Jihnhee Yu, Albert Vexler

**Affiliations:** Department of Biostatistics, University at Buffalo, 706 Kimball Tower, 3435 Main Street, Buffalo, NY 14214-3000, USA

## Abstract

We develop a new and novel exact permutation test for prespecified correlation structures such as compound symmetry or spherical structures under standard assumptions. The key feature of the work contained in this note is the distribution free aspect of our procedures that frees us from the standard and sometimes unrealistic multivariate normality constraint commonly needed for other methods.

## 1. Introduction

Let (**X**_1_,**X**_2_, …, **X***_n_*) be an iid *p*-dimensional multivariate sample from an absolutely continuous distribution *F* with *p* × *p* dispersion matrix of **X** as

(1) $$\sum =\left(\begin{array}{cccc} \sigma_{x_{1}}^{2} & \rho_{12}\sigma_{x_{1}}\sigma_{x_{2}} & \cdots & \rho_{1p}\sigma_{x_{1}}\sigma_{x_{p}}\\ \rho_{21}\sigma_{x_{2}}\sigma_{x_{1}} & \sigma_{x_{2}}^{2} & \cdots & \rho_{2p}\sigma_{x_{2}}\sigma_{x_{p}}\\ \vdots & \; & \ddots & \vdots \\ \rho_{p1}\sigma_{x_{p}}\sigma_{x_{1}} & \cdots & \cdots & \sigma_{x_{p}}^{2} \end{array}\right).$$

Inference about the dispersion matrix **Σ** takes the general form

(2) $$\begin{array}{l} H_{0}:\sum =\sum_{0},\\ H_{1}:\sum \neq \sum_{0}, \end{array}$$

where we assume that **Σ**_0_ is specified in a particular manner, for example, a block diagonal matrix or a spherical type structure or simply an unstructured form.

In general, research and testing methods of this form assume an underlying multivariate normal distribution with associated exact and approximate tests; for example, for a thorough overview and history of this testing problem, see Seber [1] and the references there within. In practice one can safely say that it would be rare that the multivariate normality assumption holds. Hence, we were motivated to develop an exact permutation method approach to this problem. To the best of our knowledge no so-called exact permutation tests have been developed or explored with the exception of the very special case of *p* = 2 dimensions and testing *H*_0_ : *ρ*_12_ = 0; for example, see Good [2]. Martin [3] provides a bootstrap algorithm for testing *H*_0_ : *ρ*_12_ = *ρ*_12,0_, which asymptotically can be shown to have the appropriate type I error rate. Unfortunately, the bootstrap methods given by Martin [3] relative to first standardizing the variables and rotating the data so as to transform the problem to the setting of testing *H*_0_ : *ρ*_12_ = 0 do not work in the permutation setting. The permutation test for the case *H*_0_ : *ρ*_12_ = 0 follows by permuting the second column of the *n* × 2 data matrix (**X**_1_,**X**_2_) and calculating the test statistic *ρ̂*_12_, where *ρ̂*_12_ refers to the standard sample Pearson correlation coefficient, over all permutations. This can be done directly via a computationally expensive algorithm or via the more widely used Monte Carlo techniques. With respect to the Monte Carlo methods we generate *B* random permutations of the data and denote the permuted value of the test statistic by ${\hat{\rho }}_{12}^{*}$. Then the one-sided *P* value for the alternative *H*_1_ : *ρ*_12_ > 0 is given as $\sum_{i=1}^{B}I({\hat{\rho }}_{12,i}^{*}\geq {\hat{\rho }}_{12})/B$, where the index *i* corresponds to a given permutation and *I* denotes the indicator function. Alternative approaches found in software packages such as SAS PROC FREQ (SAS version 9.3, Cary, NC) utilize hypergeometric probabilities similar to how Fisher’s exact test is carried out via treating the fixed data as discrete.

In general permutation testing is most often used for comparing two groups in the context of location differences or other features of distributions such as scale measures. Most of the theoretical work has been done in this setting such as type I error control. For a technical treatment of permutation testing see Romano [4] with respect to a theoretical examination for the behavior of the type I error control for permutation tests under exchangeability versus nonexchangeability conditions. In order to ensure true bounded type I error control in the permutation testing setting either the null hypothesis has to be specified in such a way that exchangeability holds by definition under *H*_0_ or some design feature such as randomization or matching needs to be employed. Commenges [5] studies the more general transformation approach used to preserve exchangeability. Also, see Zhang [6], Huang et al. [7], and Janssen and Pauls [8] with respect to the inflation of type I error rates when comparing means in the two-sample setting along with other types of comparisons. In terms of permutation testing related to correlation structure based hypotheses very little has been accomplished. This paper represents one of the only investigations of this type to date.

In Section 2 we develop the general *p*-dimensional exact tests given a prespecified covariance structure. Special cases include testing for sphericity, compound symmetry, and block diagonality, to name a few. This presentation is followed by a simulation study in Section 3. We then apply our method in Section 4 to an example involving repeated measures mice weight data.

## 2. Exact Tests for Covariance Structures in *p* Dimensions

The focus of the work in this setting is with respect to two-sided alternatives. In certain instances a subset of these tests with one-sided alternative structure may be constructed. Those tests will not be included as part of this discussion due to the specificity of their applications.

### 2.1. Unequal Variance Setting

Now let (**X**_1_,**X**_2_, …, **X***_n_*) be an iid *p*-dimensional multivariate sample from an absolutely continuous distribution *F* with the first two finite central moments corresponding to each component of **X** given as *E*(*X_i_*) = *μ*_*x*_*i*__ and $\text{Var}(X_{i})=\sigma_{x_{i}}^{2}$, *i* = 1, 2, …, *p*. Let Corr(*X_i_*, *X_j_*) = *ρ_ij_*, *i* = 1, 2, …, *p*, *j* = 1, 2, …, *p*, (*i* ≠ *j*). Furthermore, denote the *p* × *p* dispersion matrix of **X** by

(3) $$\sum =\left(\begin{array}{cccc} \sigma_{x_{1}}^{2} & \rho_{12}\sigma_{x_{1}}\sigma_{x_{2}} & \cdots & \rho_{1p}\sigma_{x_{1}}\sigma_{x_{p}}\\ \rho_{21}\sigma_{x_{2}}\sigma_{x_{1}} & \sigma_{x_{2}}^{2} & \cdots & \rho_{2p}\sigma_{x_{2}}\sigma_{x_{p}}\\ \vdots & \; & \ddots & \vdots \\ \rho_{p1}\sigma_{x_{p}}\sigma_{x_{1}} & \cdots & \cdots & \sigma_{x_{p}}^{2} \end{array}\right),$$

where **Σ** is defined to be a *p* × *p* positive definite matrix. We represent the Cholesky decomposition of the *p* × *p* matrix **Σ** as

(4) $$\sum =A^{\prime }A,$$

such that **A**′^′1^ is defined. The Cholesky decomposition is a key component of the permutation test we propose; however it is not unique to the problem; that is, other decomposition methods may yield similar results and alternative solutions. From a practical standpoint the Cholesky decomposition is built in to several statistical softwarepackages, thus making our methodology more feasible for a larger group of practitioners.

Now our more general hypothesis of interest takes the form

(5) $$\begin{array}{l} H_{0}:\sum =\sum_{0},\\ H_{1}:\sum \neq \sum_{0}, \end{array}$$

where **Σ**_0_ are the hypothesized value of **Σ** at (3).

#### Test Statistic

Let the *p* × *p* matrix $A_{0}^{-1}$ denote the transpose of **A**′^−1^ with the hypothesized values as given elements from (5). Let the *n* × *p* matrix $Z=A_{0}^{\prime -1}X$ denote the data matrix following transformation. Then the dispersion matrix corresponding to the *n* × *p* matrix **Z** will be a diagonal matrix such that Corr(*Z_i_*, *X_j_*) = 0 ∀*i* < *j*, *j* < *i* if and only if *H*_0_ at (5) holds true. Under these conditions testing *H*_0_ at (5) is equivalent to the test:

(6) $$\begin{array}{l} H_{0}:\sum_{z}=\sum_{z_{0}},\\ H_{1}:\sum_{z}\neq \sum_{z_{0}}, \end{array}$$

where the off-diagonal elements of **Σ**_*z*_0__ are equal to 0 under *H*_0_ at (6).

An exact *α*-level permutation test of *H*_0_ can be defined for (6) by considering the permutation of each column of **Z** and employing the Pearson correlation coefficient for each combination of columns. Towards this end let us denote the Corr(*Z_i_*, *Z_j_*) = *ρ*_*z*_*ij*__ with the corresponding Pearson estimator by *ρ̂*_*z*_*ij*__. The test statistic of interest in the two-sided case with respect to detecting departures from *H*_0_ at (6) is defined as

(7) $$T\;(Z)=\sum_{i=1}^{p-1}\sum_{j=i+1}^{p}\left|{\hat{\rho }}_{z_{ij}}\right|.$$

The exactness of the test in terms of the type I error control follows from a straightforward generalization of the form of the dispersion matrix for the 2 × 2 case, where

(8) $$\begin{array}{ccc} H_{0}:\rho =\rho_{0}, & \sigma_{x_{1}}=\sigma_{x_{1_{0}}}, & \sigma_{x_{2}}=\sigma_{x_{2_{0}}},\\ H_{1}:\rho \neq \rho_{0}, & \sigma_{x_{1}}\neq \sigma_{x_{1_{0}}}, & \sigma_{x_{2}}\neq \sigma_{x_{2_{0}}}. \end{array}$$

In the 2 × 2 case the off-diagonal elements of the dispersion matrix are given as

(9) $$\sum_{Z|H_{1}}=\left(\begin{array}{cc} \frac{\sigma_{x_{1}}^{2}}{\sigma_{x_{1_{0}}}^{2}} & \frac{\sigma_{x_{1}}\;\left(\rho \sigma_{x_{1_{0}}}\sigma_{x_{2}}-\rho_{0}\sigma_{x_{1}}\sigma_{x_{2_{0}}}\right)}{\sqrt{1-\rho_{0}}\sigma_{x_{1_{0}}}^{2}\sigma_{x_{2_{0}}}^{2}}\\ \frac{\sigma_{x_{1}}\;\left(\rho \sigma_{x_{1_{0}}}\sigma_{x_{2}}-\rho_{0}\sigma_{x_{1}}\sigma_{x_{2_{0}}}\right)}{\sqrt{1-\rho_{0}}\sigma_{x_{1_{0}}}^{2}\sigma_{x_{2_{0}}}^{2}} & \frac{\sigma_{x_{1_{0}}}^{2}\sigma_{x_{2}}^{2}-2\rho \rho_{0}\sigma_{x_{1}}\sigma_{x_{2}}\sigma_{x_{1_{0}}}\sigma_{x_{2_{0}}}+\rho_{0}^{2}\sigma_{x_{1}}^{2}\sigma_{x_{2_{0}}}^{2}}{(1-\rho_{0})\;\sigma_{x_{1_{0}}}^{2}\sigma_{x_{2_{0}}}^{2}} \end{array}\right).$$

An examination of the covariance term corresponding to **Σ_Z_**_|*H*_1__ at (9) clearly indicates that it has the value of 0 if and only if *H*_0_ at (8) is true. When testing *H*_0_ at (8) the Pearson correlation estimate between the transformed variates through $A_{0}^{-1}$, *Z*_1_, and *Z*_2_ serves to appropriately detect departures from *H*_0_. Within the permutation testing framework provides an exact *α*-level test; that is, the covariance of *Z*_1_ and *Z*_2_ is 0 if and only if the correlation of *Z*_1_ and *Z*_2_ is 0.

We resort to a Monte Carlo approximation in order to obtain the *P* value for testing *H*_0_ defined at (5). The steps for performing the Monte Carlo approximation with respect to estimating the *P* value are as follows.

1. Define *H*_0_ at (5).
2. Obtain the new random variates **Z** by applying the transformation $Z=A_{0}^{\prime -1}X$ to the observed data **X**.
3. Calculate *T*(**Z**) at (7).
4. Permute each column of **Z** independently such that we have the permuted *n* × *p* matrix denoted by Z^*^.
5. Calculate *T*(Z^*^) applying the resampled values to *T*(**Z**) at (7).
6. Repeat steps (4) and (5) *B* times.
7. Calculate the Monte Carlo estimated permutation *P* value as $p=(\sum_{l=1}^{B}I_{\left(T{\left(Z^{*}\right)}_{l}>T(Z)\right)})/B$, where *I*_(·)_ denotes the indicator function.

### 2.2. Nontransformation Special Cases

In certain special cases we can test specific forms of hypothesis (5) using our permutation approach without specifying a specific subset of the $\sigma_{x_{i}}^{2}$’s or *ρ_ij_*’s. One obvious special case relative to testing hypothesis (5) is the test given for a diagonal dispersion matrix versus nondiagonal dispersion matrix such that under *H*_0_ all *ρ_ij_* = 0. Historical tests of this form have relied on assuming *p*-variate multivariate normality; for example, see Mudholkar et al. [9] for a description of a likelihood ratio approximation to this test. In this instance we have

(10) $$\sum_{0}=\left(\begin{array}{cccc} \sigma_{x_{1}}^{2} & 0 & \cdots & 0\\ 0 & \sigma_{x_{2}}^{2} & \cdots & 0\\ \vdots & \; & \ddots & \vdots \\ 0 & \cdots & \cdots & \sigma_{x_{p}}^{2} \end{array}\right),$$

with unspecified $\sigma_{x_{i}}^{2}$’s under *H*_0_.

In this case there is no transformation of the data required. An exact *α*-level permutation test of *H*_0_ can be defined simply by considering the permutation of each column of **X** and employing the Pearson correlation coefficient for each combination of columns. Towards this end denote the Corr(*X_i_*, *X_j_*) = *ρ_ij_* with the corresponding Pearson estimator by *ρ̂*_*x*_*ij*__. The test statistic of interest with respect to detecting departures from the diagonal structure is defined as

(11) $$T\;(X)=\sum_{i=1}^{p-1}\sum_{j=i+1}^{p}\left|{\hat{\rho }}_{x_{ij}}\right|.$$

The Monte Carlo estimated permutation *P* value is calculated similarly as before, where $p=(\sum_{l=1}^{B}I_{\left(T{\left(X^{*}\right)}_{l}>T(X)\right)})/B$, where *I*_(·)_ denotes the indicator function.

Another special case where we can have a set of unspecified $\sigma_{x_{i}}^{2}$’s or *ρ_ij_*’s is when we may be interested in testing for a block diagonal dispersion matrix structure such that under *H*_0_ at (5) we now have

(12) $$\sum_{0}=\left(\begin{array}{cccc} \sum_{11} & 0 & \cdots & 0\\ 0 & \sum_{22} & \cdots & 0\\ \vdots & \; & \ddots & \vdots \\ 0 & \cdots & \cdots & \sum_{\mathrm{bb}} \end{array}\right),$$

where the partitioned *q_i_* × *q_j_* matrices are given as

(13) $$\sum_{\mathrm{jj}}=\left(\begin{array}{cccc} \sigma_{x_{1}}^{2} & \rho_{12}\sigma_{x_{1}}\sigma_{x_{2}} & \cdots & \rho_{1q}\sigma_{x_{1}}\sigma_{x_{q}}\\ \rho_{21}\sigma_{x_{2}}\sigma_{x_{1}} & \sigma_{x_{2}}^{2} & \cdots & \rho_{2q}\sigma_{x_{2}}\sigma_{x_{q}}\\ \vdots & \; & \ddots & \vdots \\ \rho_{q1}\sigma_{x_{q}}\sigma_{x_{1}} & \cdots & \cdots & \sigma_{x_{q}}^{2} \end{array}\right).$$

The dispersion matrix **Σ_jj_** may have different dimensions *q_j_* < *p*(Σ*q_i_* = *p*), *j* = 1, 2, …, *b*, with unspecified $\sigma_{x_{i}}^{2}$’s and *ρ_ij_*’s under *H*_0_.

As in the test for a diagonal dispersion matrix above there is no transformation of the data required. An exact *α*-level permutation test of *H*_0_ can again be defined simply by considering the permutation of each column of **X** and employing the Pearson correlation coefficient for each combination of columns. The test statistic of interest with respect to detecting departures from the diagonal structure is a slight modification of the test statistic at (11) defined as

(14) $$T\;(X)=\sum_{i=1}^{p-1}\sum_{j=i+1}^{p}\left|{\hat{\rho }}_{x_{ij}}\right|I_{\left(\rho_{0,ij}=0\right)},$$

where the “off-block” correlation elements at (12), *ρ*_0,_*_ij_* = 0, under *H*_0_ and *I*_(·)_ denote the indicator function. The Monte Carlo estimated permutation *p* value is calculated similarly as before, where $p=(\sum_{l=1}^{B}I_{\left(T{\left(X^{*}\right)}_{l}>T(X)\right)})/B$ and *I*_(·)_ denotes the indicator function.

### 2.3. Equal Variance Setting

For the equal variance *p*-dimensional case we have

(15) $$\begin{array}{l} H_{0}:\sum =\sum_{0},\\ H_{1}:\sum \neq \sum_{0}, \end{array}$$

where we now define the *p* × *p* dispersion matrix under *H*_0_ as

(16) $$\sum_{0}=\sigma \Gamma_{0},$$

where **Σ**_0_ is defined to be a *p* × *p* positive definite matrix, Var(**X***_i_*) = *σ*^2^, *i* = 1, 2, …, *p*, under *H*_0_ and the *p* × *p* correlation matrix **Γ**_0_ is given by

(17) $$\Gamma_{0}=\left(\begin{array}{cccc} 1 & \rho_{12,0} & \cdots & \rho_{1p,0}\\ \rho_{21,0} & 1 & \cdots & \rho_{2p,0}\\ \vdots & \ddots & \vdots & \;\\ \rho_{p1,0} & \cdots & \cdots & 1 \end{array}\right).$$

The Cholesky decomposition of matrix **Γ**_0_ is as

(18) $$\Gamma_{0}=B_{0}^{\prime }B_{0}.$$

In order to test the hypothesis at (15) we utilize the transformation $Z=B_{0}^{\prime -1}X$. Note that *H*_0_ at (15) will be sensitive to departures from **Γ**_0_ and unequal marginal variances. Furthermore, explicit values for *σ*^2^ do not need to be specified within this hypothesis testing framework.

The steps for performing the Monte Carlo approximation with respect to estimating the *p* value are as follows.

1. Define *H*_0_ at (15).
2. Obtain the new random variates **Z** by applying the transformation $Z=B_{0}^{\prime -1}X$ to the observed data **X**.
3. Calculate *T*(**Z**) at (7).
4. Permute each column of **Z** independently such that we have the permuted *n* × *p* matrix denoted by Z^*^.
5. Calculate *T*(Z^*^).
6. Repeat steps (4) and (5) *B* times.
7. Calculate the Monte Carlo estimated permutation *P* value as $p=(\sum_{l=1}^{B}I_{\left(T{\left(Z^{*}\right)}_{l}>T(Z)\right)})/B$, where *I*_(·)_ denotes the indicator function.

A special case relative to testing hypothesis (15) is the test that the dispersion matrix is diagonal and all *σ*_*x*_*i*__ = *σ*, *i* = 1, 2, …, *p*.

Other special cases of the test at (15) may be of interest and written in the form

(19) $$\begin{array}{l} H_{0}:\sum =\sigma \Gamma_{0}\;(\rho_{0}),\\ H_{1}:\sum \neq \sigma \Gamma_{0}\;(\rho_{0}). \end{array}$$

Examples of specific dispersion structures of importance corresponding to the test at (19) include

1. sphericity:
  (20) $$\Gamma_{0}\;(\rho_{0})=\left(\begin{array}{ccccc} 1 & 0 & \cdots & 0 & 0\\ 0 & 1 & \cdots & 0 & 0\\ \vdots & \vdots & \ddots & \vdots & \vdots \\ 0 & 0 & \cdots & 1 & 0\\ 0 & 0 & \cdots & 0 & 1 \end{array}\right);$$
2. compound symmetry:
  (21) $$\Gamma_{0}\;(\rho_{0})=\left(\begin{array}{ccccc} 1 & \rho_{0} & \cdots & \rho_{0} & \rho_{0}\\ \rho_{0} & 1 & \cdots & \rho_{0} & \rho_{0}\\ \vdots & \vdots & \ddots & \vdots & \vdots \\ \rho_{0} & \rho_{0} & \cdots & 1 & \rho_{0}\\ \rho_{0} & \rho_{0} & \cdots & \rho_{0} & 1 \end{array}\right);$$
3. first-order autoregressive:
  (22) $$\Gamma_{0}\;(\rho_{0})=\left(\begin{array}{ccccc} 1 & \rho_{0} & \cdots & \rho_{0}^{p-1} & \rho_{0}^{p}\\ \rho_{0} & 1 & \cdots & \rho_{0}^{p-2} & \rho_{0}^{p-1}\\ \vdots & \vdots & \ddots & \vdots & \vdots \\ \rho_{0}^{p-1} & \rho_{0}^{p-2} & \cdots & 1 & \rho_{0}\\ \rho_{0}^{p} & \rho_{0}^{p-1} & \cdots & \rho_{0} & 1 \end{array}\right);$$
4. spatial power:
  (23) $$\Gamma_{0}\;(\rho_{0})=\left(\begin{array}{ccccc} 1 & \rho_{0}^{d_{1,2}} & \cdots & \rho_{0}^{d_{1,p-1}} & \rho_{0}^{d_{1,p}}\\ \rho_{0}^{d_{2,1}} & 1 & \cdots & \rho_{0}^{d_{2,p-2}} & \rho_{0}^{d_{2,p-1}}\\ \vdots & \vdots & \ddots & \vdots & \vdots \\ \rho_{0}^{d_{p-1,1}} & \rho_{0}^{d_{p-1,2}} & \cdots & 1 & \rho_{0}^{d_{p-1,p}}\\ \rho_{0}^{d_{p,1}} & \rho_{0}^{d_{p,2}} & \cdots & \rho_{0}^{d_{p,p-1}} & 1 \end{array}\right).$$

Several other well-known spatial dispersion matrices similar to the spatial power matrix presented above fit within this same framework and will not be presented here.

## 3. Simulation Study

In this section we examine the test at (19), where we specify *ρ*_0_ and the form of the correlation structure **Γ** at (17), for example, compound symmetry. Our simulation study for the *p* × *p* case will utilize a *p*-variate standardized multivariate normal distribution with *p* = 5 and a special case mixing the marginal distributions across normal, exponential, and uniform forms. Again, differing location and scale doe not vary the general conclusions. In terms of our simulation study we set the null value of *ρ*_0_ = 0, 0.5, 0.9 under a compound symmetry assumption and a first-order autoregressive assumption, where **Γ** will take the forms:

1. compound symmetry:
  (24) $$\Gamma_{0}\;(\rho_{0})=\left(\begin{array}{ccccc} 1 & \rho_{0} & \cdots & \rho_{0} & \rho_{0}\\ \rho_{0} & 1 & \cdots & \rho_{0} & \rho_{0}\\ \vdots & \vdots & \ddots & \vdots & \vdots \\ \rho_{0} & \rho_{0} & \cdots & 1 & \rho_{0}\\ \rho_{0} & \rho_{0} & \cdots & \rho_{0} & 1 \end{array}\right);$$
2. first-order autoregressive:
  (25) $$\Gamma_{0}\;(\rho_{0})=\left(\begin{array}{ccccc} 1 & \rho_{0} & \cdots & \rho_{0}^{p-1} & \rho_{0}^{p}\\ \rho_{0} & 1 & \cdots & \rho_{0}^{p-2} & \rho_{0}^{p-1}\\ \vdots & \vdots & \ddots & \vdots & \vdots \\ \rho_{0}^{p-1} & \rho_{0}^{p-2} & \cdots & 1 & \rho_{0}\\ \rho_{0}^{p} & \rho_{0}^{p-1} & \cdots & \rho_{0} & 1 \end{array}\right).$$

Note that the special case *ρ*_0_ = 0 is the same for both covariance structures and is only presented once. It should also be noted that under the assumption of multivariate normality testing *H*_0_ : **Σ** = *σ***Γ**_0_(0) under the compound symmetry or first-order autoregressive structure is a special case (equal variance assumption) of the well-known test for “complete independence”; for example, see Mudholkar et al. [9]. Under nonnormality we are essentially testing the “complete uncorrelated” case. In this special case the methods presented here are the first exact methods developed for tackling this particular hypothesis. In terms of large sample theory around similar results see Jiang [10] and Xiao and Wu [11].

**Figure 1:**
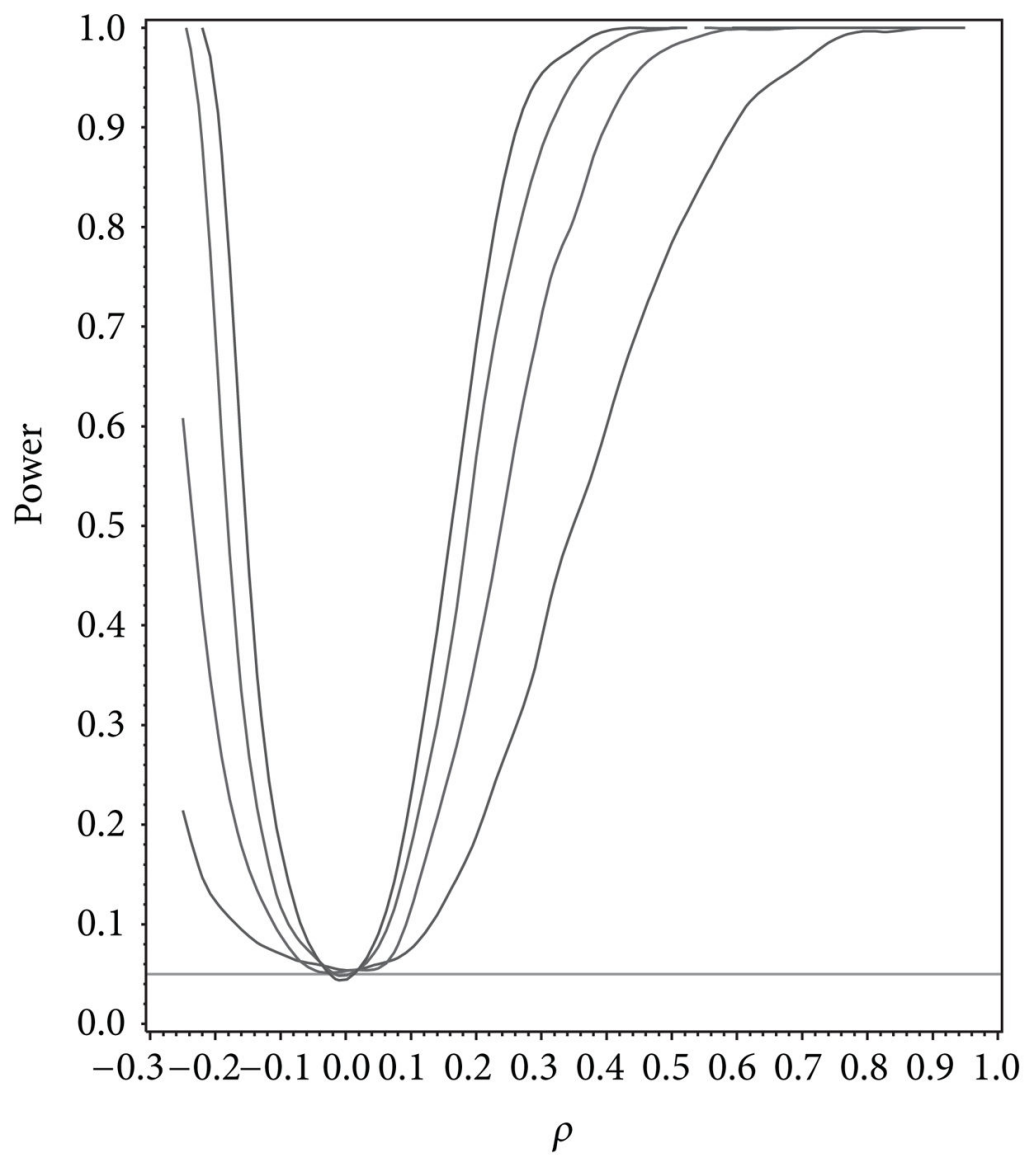
Power for testing for compound symmetry covariance as a function of *ρ* given *ρ*_0_ = 0.

**Figure 2:**
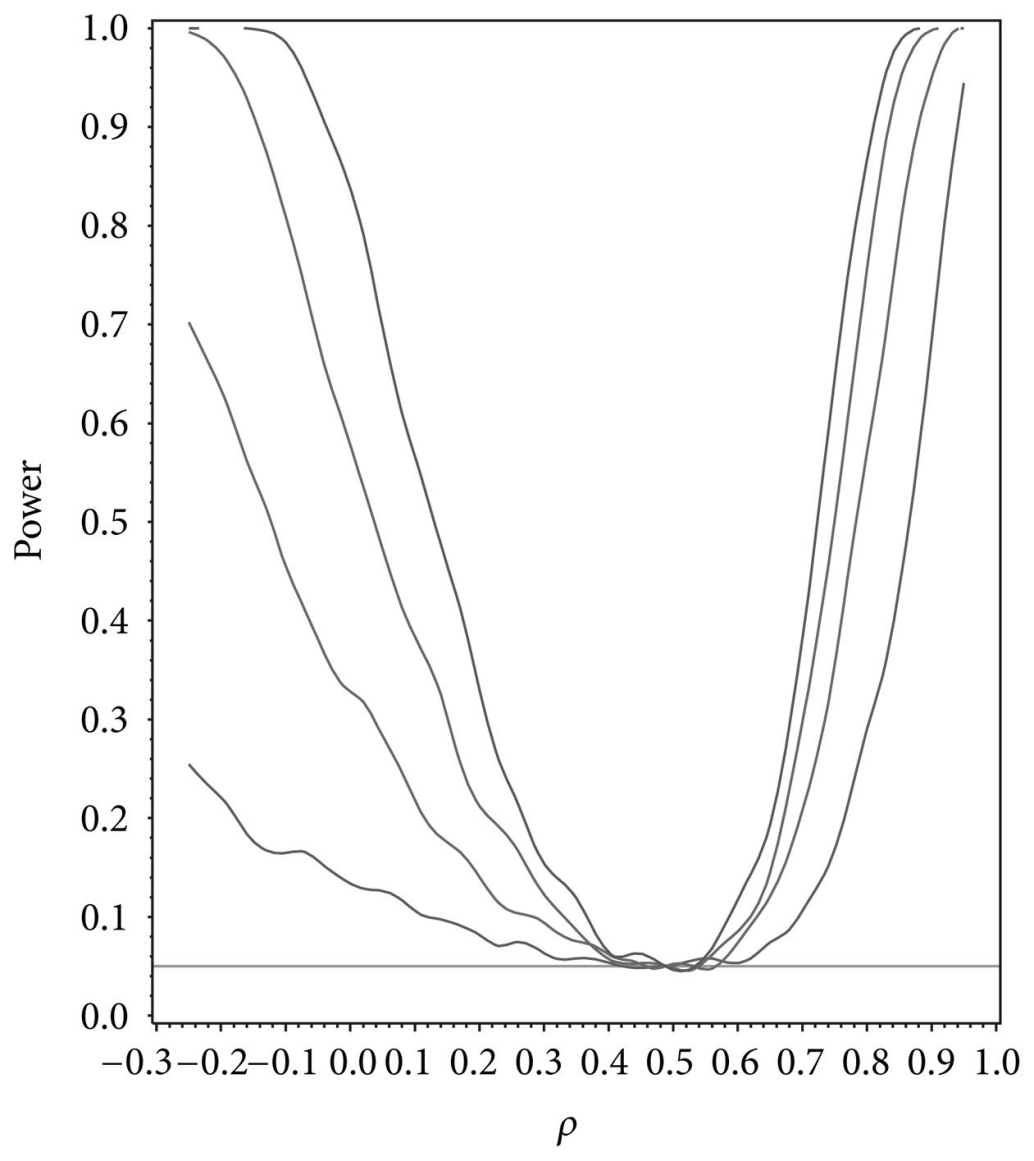
Power for testing for compound symmetry covariance as a function of *ρ* given *ρ*_0_ = 0.5.

**Figure 3:**
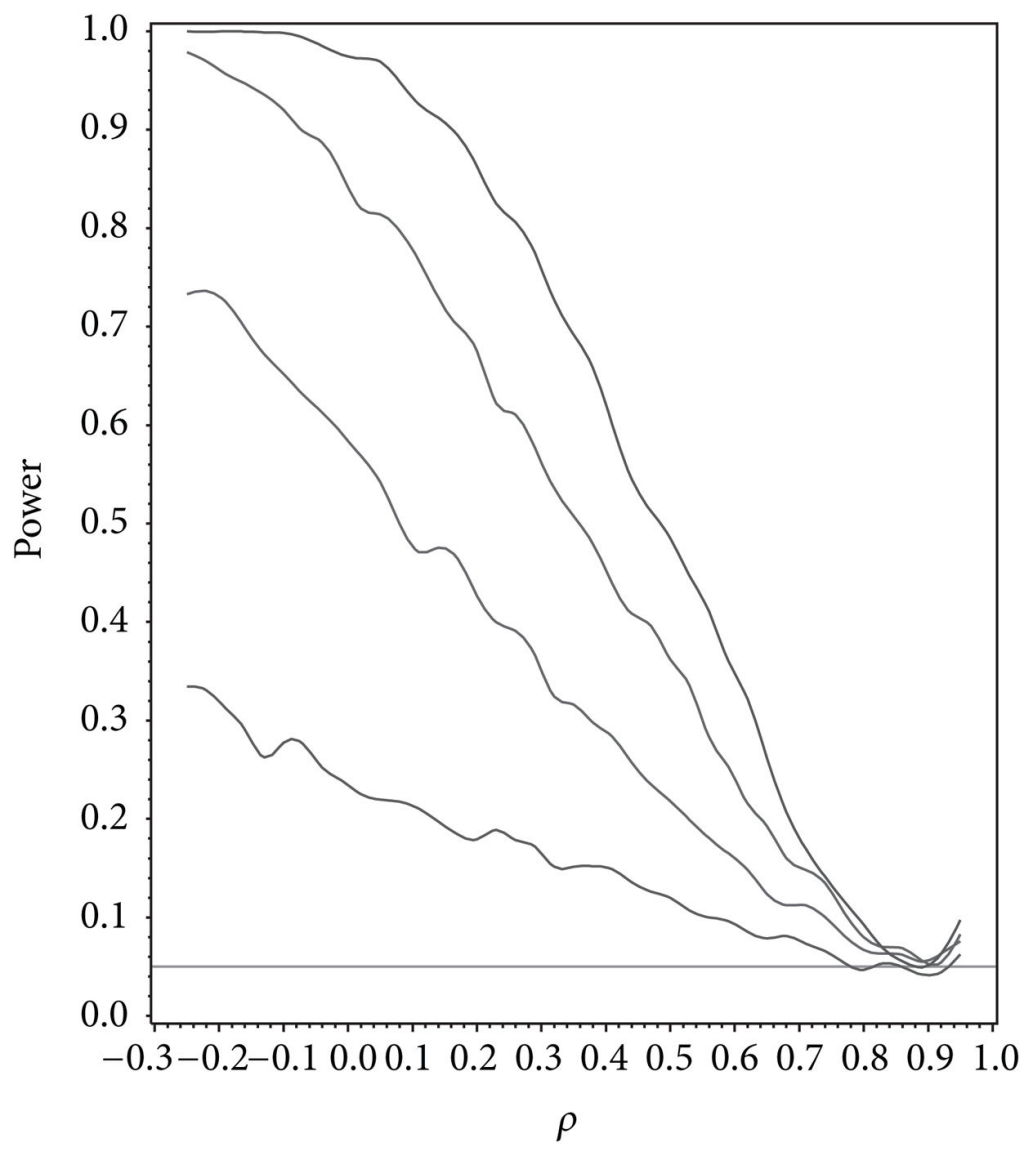
Power for testing for compound symmetry covariance as a function of *ρ* given *ρ*_0_ = 0.9.

**Figure 4:**
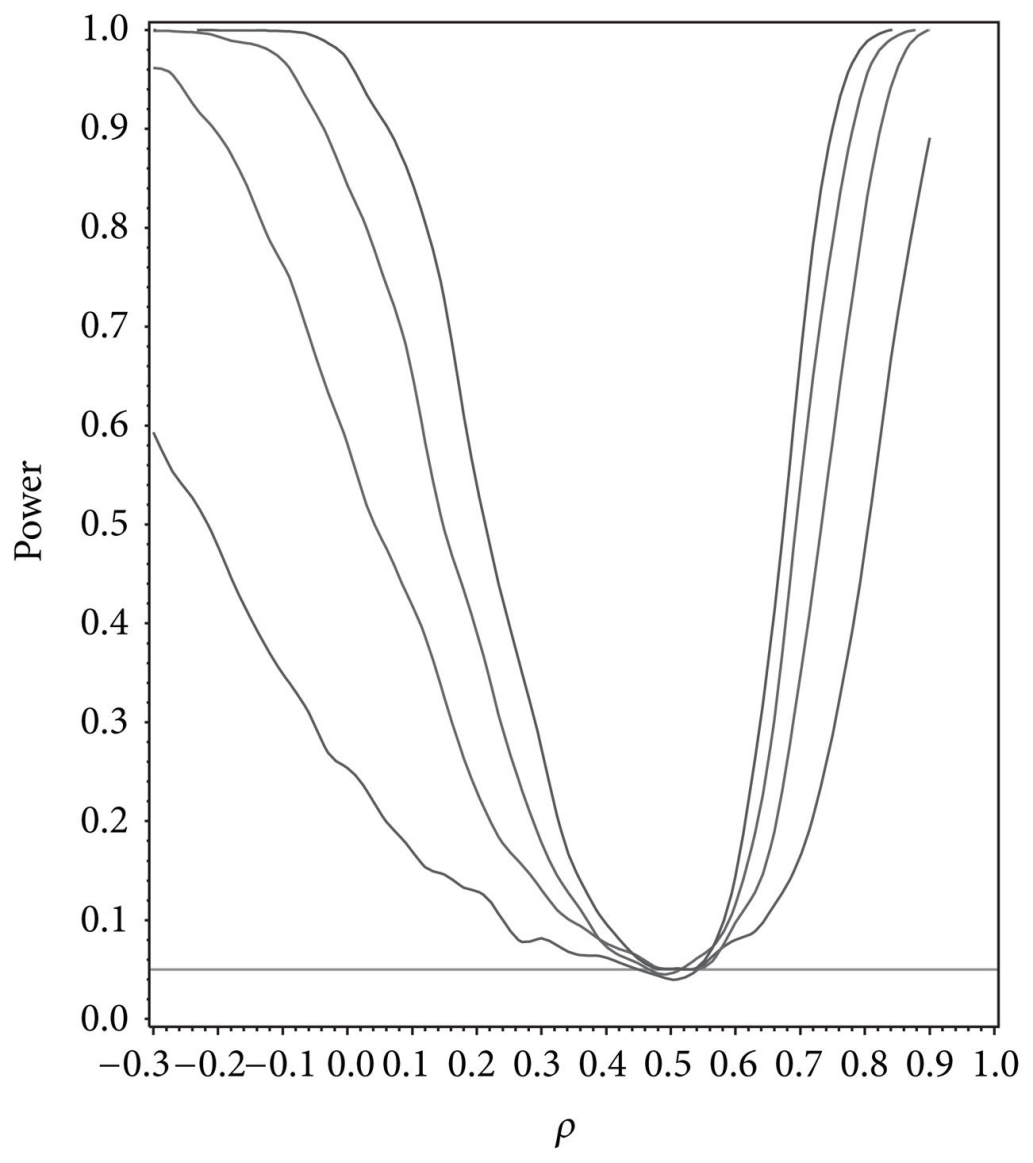
Power for testing for first-order autoregressive covariance as a function of *ρ* given *ρ*_0_ = 0.5.

**Figure 5:**
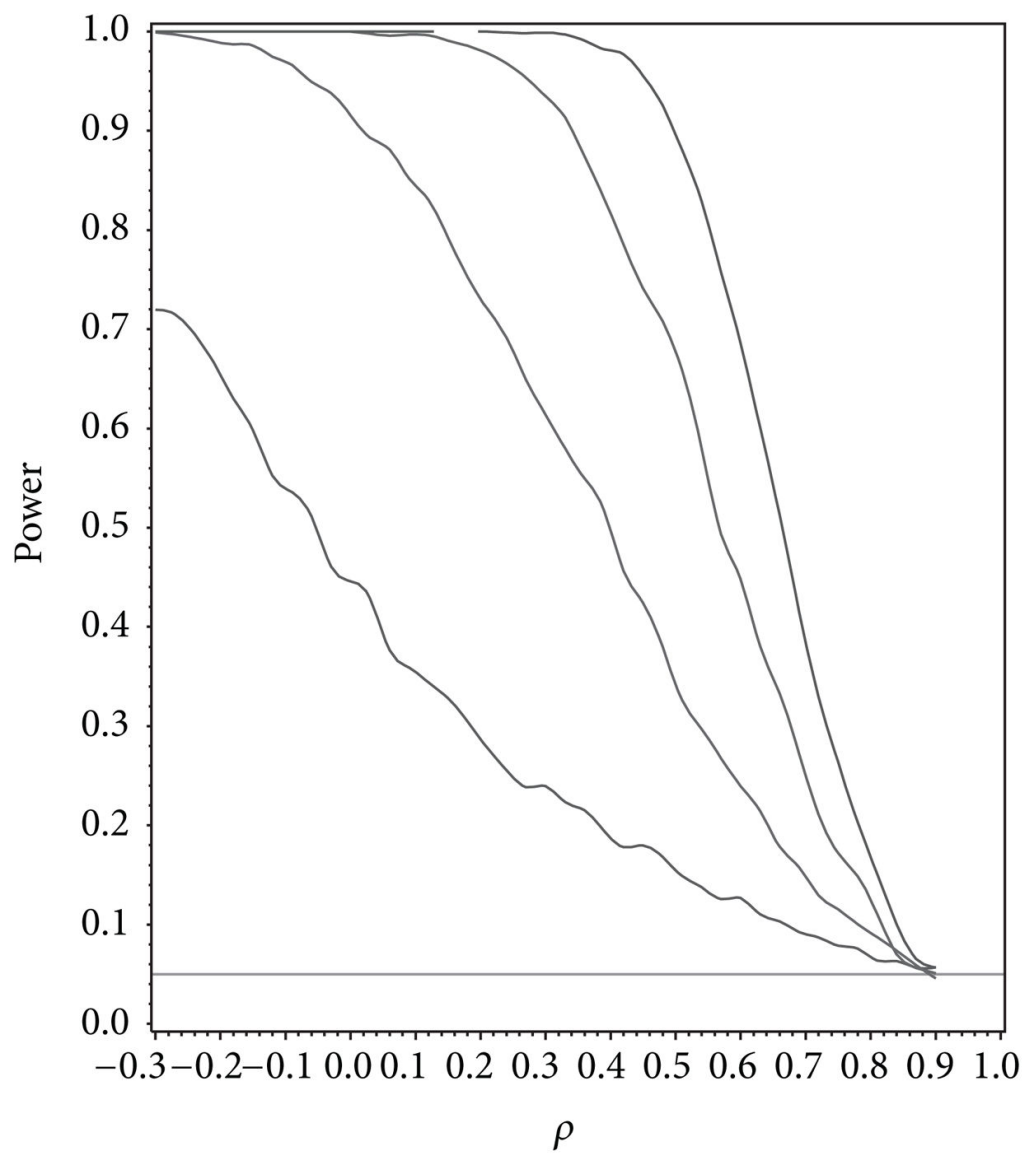
Power for testing for first-order autoregressive covariance as a function of *ρ* given *ρ*_0_ = 0.9.

For our simulation study we used 1000 replicates for our study at *n* = 10, 20, 30, 40 and set *α* = 0.05. The covariance structure was the same under *H*_0_ and *H*_1_ for this set of simulations. The results are contained in Figures 1, 2, 3, 4, and 5. As anticipated we see the expected results of appropriate type I error control and monotone power functions increasing in either direction about the null value for *ρ*. The range of *ρ* under the alternative was dictated by the constraint that **Γ**_0_(*ρ*_0_) is defined to be positive definite.

**Figure 6:**
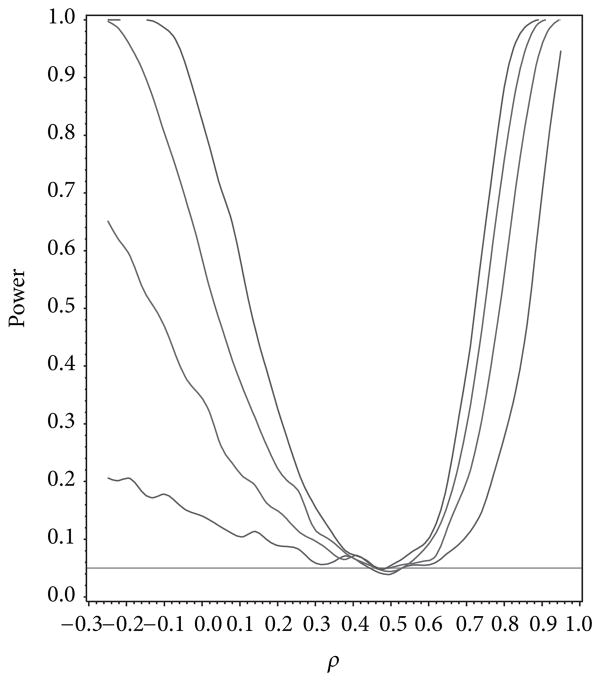
Power for testing for compound symmetry covariance as a function of *ρ* given *ρ*_0_ = 0.5 and mixed marginal distributions.

For the sake of example we modified our simulation and took *ρ*_0_ = 0.5 with marginals given by *X*_1_, *X*_2_ ~ *N*(0, 1), $X_{3},X_{4}\sim U(-1/\sqrt{12},1/\sqrt{12})$, and *X*_5_ exp(1, −1) with **Γ**_0_(*ρ*_0_) assumed to be compound symmetric under *H*_0_ and under *H*_1_. The results are shown in Figure 6 and as we can see they do not differ dramatically from Figure 2 assuming multivariate normality, thus illustrating the flexibility and nonparametric nature of our methodology.

As an additional result we studied the power under the correctly specified *ρ* under *H*_0_ with **Γ**_0_(*ρ*_0_) differing in structure. For this study we set *ρ*_0_ = 0.5, 0.9 with **Γ**_0_(*ρ*_0_) set to compound symmetry under *H*_0_ and **Γ**_0_(*ρ*_0_) set to the first-order autoregressive structure under *H*_1_. In other words what is the power to detect a correlation structure different from the null structure given that *ρ*_0_ is the true correlation. At *α* = 0.05 the power to detect a different correlation structure under the alternative for *ρ*_0_ = 0.5 and 0.9 at *n* = 10, 20, 30, 40 and *α* = 0.05 was 0.245, 0.539, 0.761, and 0.894 and 0.520, 0.858, 0.951, and 0.998, respectively.

## 4. Example

As an illustration of our method we will use phenotypic weight data from *n* = 16 mice as contained in Table 1 from a recent unpublished study conducted within Roswell Park Cancer Institute. The estimated correlation matrix is provided in Table 2. The respective sample variance estimates were ${\hat{\sigma }}_{x_{1}}^{2}=3.2,\;{\hat{\sigma }}_{x_{2}}^{2}=3.3,\;{\hat{\sigma }}_{x_{3}}^{2}=2.6,\;{\hat{\sigma }}_{x_{4}}^{2}=3.4$, and ${\hat{\sigma }}_{x_{5}}^{2}=3.1$. For example, suppose we were interested in testing

(26) $$\begin{array}{l} H_{0}:\sum =\sigma \Gamma_{0}\;(.95),\\ H_{1}:\sum \neq \sigma \Gamma_{0}\;(.95), \end{array}$$

for both Γ(·) having the compound symmetry structure or the first-order autoregressive structure as defined in (23). In this instance the test corresponding to the above hypothesis under a compound symmetry correlation structure yielded a Monte Carlo estimated *P* value <0.0001 (*B* = 10, 000). While the test corresponding to the above hypothesis under the first-order autoregressive correlation structure yielded a Monte Carlo estimated *P* value = 0.002 (*B* = 10, 000). For this example this provides some measure of evidence that the correlation structure does not fit the compound symmetry structure and that the first-order autoregressive structure assuming *ρ* = 0.95 may be more appropriate. Similarly, the test for diagonality under the equal variances assumption (sphericity), which does not assume a value for *ρ*_0_, yielded a MonteCarlo estimated *P* value <0.0001 (*B* = 10, 000). Note that we may be rejecting *H*_0_ at some specified level *α* under at least one of 3 scenarios: unequal marginal variances, *ρ* ≠ *ρ*_0_ or **Γ** ≠ **Γ**_0_.

Given our overall *P* value from above which was 0.002, we may wish to examine in further detail what is driving us to reject *H*_0_. In this case we can examine specific submatrices of the dispersion matrix of $Z=B_{0}^{\prime -1}X$. For this example we could test *H*_0_ : **Σ** = *σ***Γ**_0_(.95) using days 0, 1, and 2, only or any other combinations of days such that the appropriate correlation substructure is extracted from the original hypothesized values for **Γ**. For our example subtest we get *P* = 0.32 indicating no strong evidence against a first order autoregressive “substructure” with equal variances and *ρ*_0_ = 0.95. If we add day 3, our *P* value=0.04, indicating either the correlation structure may be misspecified at this point or the variance is different. Note that further work relative to the multiple comparison problem of subtests and their relative correlation is needed. This is simply an exploratory approach to this issue relative to the example at hand.

## 5. Concluding Remarks

In this note we provided a method for exact testing around specific covariance structures. We employed the Cholesky decomposition for this purpose. It was noted by a reviewer that other decomposition methodologies may lead to extensions of this methodology, which we will consider in terms of future work.

## Figures and Tables

**Table 1:** Mice weights (grams).

Mouse	Day 0	Day 1	Day 2	Day 3	Day 4
1	21.4	21.0	21.0	21.3	21.5
2	18.4	18.4	18.0	18.1	17.8
3	17.8	17.6	17.4	17.2	17.1
4	18.2	18.5	17.6	18.0	17.5
5	20.0	19.4	19.1	19.6	18.9
6	20.2	19.3	19.3	19.3	18.6
7	16.5	16.3	16.6	16.4	16.3
8	17.6	17.4	17.5	17.9	17.6
9	19.8	20.4	20.1	20.6	20.3
10	22.0	22.3	21.3	21.9	20.7
11	17.5	17.4	17.5	17.5	17.3
12	20.3	20.2	19.8	20.4	19.7
13	16.3	16.2	16.2	15.9	15.7
14	18.0	17.2	17.2	17.0	16.4
15	20.3	19.9	19.0	18.9	18.1
16	21.4	21.2	20.7	21.1	20.7

**Table 2:** Estimated correlation matrix for mouse weight example data.

	Day 0	Day 1	Day 2	Day 3	Day 4
Day 0	1.000	0.977	0.974	0.959	0.921
Day 1	0.977	1.000	0.982	0.979	0.945
Day 2	0.974	0.982	1.000	0.993	0.979
Day 3	0.959	0.979	0.993	1.000	0.983
Day 4	0.921	0.945	0.979	0.983	1.000
